# Life history response of *Echinops gmelinii* Turcz. to variation in the rainfall pattern in a temperate desert

**DOI:** 10.7717/peerj.8159

**Published:** 2019-11-29

**Authors:** Yanli Wang, Xinrong Li, Lichao Liu, Jiecai Zhao, Jingyao Sun

**Affiliations:** 1 Shapotou Desert Research and Experimental Station, Northwest Institute of Eco-Environment and Resources, Chinese Academy of Sciences, Lanzhou, China; 2 University of Chinese Academy of Sciences, Beijing, China

**Keywords:** Climate change, Rainfall pattern, *Echinops gmelinii* Turcz., Winter annual, Life history, Temperate desert

## Abstract

**Background:**

Current and future changes in rainfall amount and frequency may particularly impact annual plants in desert ecosystems. The winter annual *Echinops gmelinii* Turcz. is widely distributed in the desert habitats of northern China and is a dominant pioneer annual plant following sand stabilization in the Tengger Desert. This species plays a vital role in dune stabilization during spring and early summer, when wind erosion is the most severe and frequent. However, seedling emergence and regeneration in sandy soil are mainly determined by rainfall patterns. Therefore, understanding the life history response of this species to rainfall variation is necessary for understanding the change of population dynamics under the future climate change.

**Methods:**

A field simulation rainfall pot experiment using rainout shelter was conducted that included five amounts and five frequencies of rainfall based on historical and predicted values to monitor the life history responses of *E. gmelinii* in a near-natural habitat.

**Results:**

We found that rainfall amount and frequency significantly affected seedling survival, growth and reproduction. The plant height, biomass, capitula number, seed number, seed mass and reproductive effort, but not the root/shoot ratio, significantly increased with increasing rainfall. Further, these traits exhibited the greatest response to low-frequency and larger rainfall events, especially the optimal rainfall frequency of 10-day intervals. Offspring seed germination showed increasing trends with decreasing rainfall, suggesting that the maternal effects may have occurred.

**Conclusions:**

Our study shows that the plasticity in growth and reproduction of *E. gmelinii* in response to rainfall variations may help it to gain dominance in the harsh and unpredictable desert environment. Furthermore, population development of this winter annual species should be promoted under the likely future scenarios of large rainfall events and increasing cool-season precipitation in temperate desert.

## Introduction

Global climate change is predicted to further increase variation in rainfall, with more extreme rainfall events punctuated by longer intervening dry periods and changes in seasonality (IPCC, 2013). These shifts in rainfall should have greater effects on plant community composition in arid and semiarid ecosystems, where precipitation is scarce and there is high inter- and intra-annual variability (Noy-Meir, 1973; Muldavin et al., 2008; Báez et al., 2013; Chen et al., 2019b). Specifically, rainfall fluctuation is known to cause particularly high variation in the populations of annual plants (Went, 1948; Beatley, 1974; Young et al., 1981; Angert et al., 2007; Levine, Mceachern & Cowan, 2008). Long-term monitoring of a winter annual plant community demonstrated that demographic success is strongly related to growing season precipitation, but species have also been found to differ in the degree of demographic sensitivity to precipitation (Venable, 2007; Huxman et al., 2008). Furthermore, Miranda et al. (2009) showed that higher reductions or long-term changes in water availability would likely reduce productivity and diversity in three semiarid plant communities dominated by annual species in Mediterranean ecosystems. Plant communities respond not only to the rainfall amount but also to variation in timing, especially for annual species in arid environments, where relatively small changes in rainfall frequency may have strong effects on communities (Sala & Lauenroth, 1982; Knapp et al., 2002; Miranda et al., 2011). In the southwestern United States, less frequent and larger rainfall events could provide a competitive advantage to *Bouteloua gracilis* and influence species composition in the arid-semiarid grassland ecotone (Thomey et al., 2014). Despite the existence of many experimental studies demonstrating links between rainfall regimes and ecological processes, the understanding of the plant species response to variation in rainfall patterns at the regional scale is still inadequate (Miranda, Padilla & Pugnaire, 2009; Thomey et al., 2014; Gao et al., 2015; Mojzes et al., 2018; March-Salas & Fitze, 2019).

Phenotypic plasticity is one of the key mechanisms that can allow plant populations to adjust to climate change (Nicotra et al., 2010; Franks, Weber & Aitken, 2014; Parmesan & Hanley, 2015). Some previous studies have examined the plastic responses of plant physiology (Liu, Zhao & Wen, 2012; Thomey et al., 2014), seed germination and seedling survival, growth and reproduction (Pol, Pirk & Marone, 2010; Lu et al., 2012; Gao et al., 2015; Prado-Tarango et al., 2018) to variation in annual precipitation. However, most studies have addressed the plastic response of growth traits, such as biomass accumulation, to environmental changes (Muldavin et al., 2008; Miranda, Padilla & Pugnaire, 2009; Li, Zhao & Liu, 2015; Sepúlveda et al., 2018). The plasticity of certain regeneration traits, such as seed germination and seedling growth, is highly unknown, despite the critical role of early life-history stages in plant population persistence (Walck et al., 2011; Hanel & Tielborger, 2015). Moreover, the plastic response of an individual can be expressed not only as within-generation phenotypic plasticity but also the potential importance of maternal environmental effects on plant species’, which responses to global environmental changes has been highlighted by an increasing number of studies (Hovenden et al., 2008; Pias et al., 2010; Fenesi et al., 2014; Walter et al., 2016; Mojzes et al., 2018). Rainfall changes in the maternal environment could influence offspring germination behaviors because dormancy has been found to be broken by drought (Cendán, Sampedro & Zas, 2013; Baskin & Baskin, 2014; Chen et al., 2019b). Unfortunately, the analyses of the phenotypic responses to climate change are almost solely from the perspective of individual rainfall events or one stage of a plants’ life history, and little is known about how a series of pulse events affect the whole life cycle of annual plants.

Winter annuals contribute substantially to plant diversity in desert regions and have received a great deal of attention regarding their life history responses to climate change in tropical and Mediterranean climate regions (Huxman et al., 2008; Huxman & Venable, 2013; Dwyer & Erickson, 2016; Mojzes et al., 2018). However, little is known about the ecology of desert winter annuals in temperate zones, which are characterized by cold and dry winters (Baskin & Baskin, 2014). In the Tengger Desert, which is a typical temperate desert in northern China, *Echinops gmelinii* Turcz. is the most common winter annual species. It is widely distributed in Siberia, Mongolia and northern China and grows in sandy soil and shingle habitats (Shi, 1987). On the southeast margin of the Tengger Desert, the species is a dominant pioneer annual plant following sand stabilization, where its coverage can reach 20–30% in May, and the presence of this species plays a vital role in preventing wind erosion and maintaining sand fixation during the spring and early summer. *E. gmelinii* seeds germinate in summer and autumn, and plants overwinter as rosettes and complete their life cycle quickly by utilizing spring and early summer rainfall (Wang et al., 2019a), which could avoid resource competition with other summer annuals (Tobe, Zhang & Omasa, 2005). At a field site in the Tengger Desert, we found that the population dynamics of *E. gmelinii* were very sensitive to rainfall variation (Wang et al., 2019b); thus, the species may be particularly threatened by climate change. Furthermore, *E. gmelinii* provides an ideal opportunity to understand the response of the life history adaptation strategies of winter annuals in temperate desert to rainfall variation in the context of global climate change.

Thus, to test the effects of rainfall pattern variation on the survival, growth and reproduction of *E. gmelinii*, we established a gradient of five amounts and five frequencies of rainfall based on historical and predicted values (1955–2015). Specifically, the aims of this study were to answer the following questions: (1) How are the survivorship, growth, and reproduction of *E. gmelinii* affected by variation in rainfall pattern? (2) Do changes in the maternal environment caused by different rainfall amounts and frequencies influence the offspring seed germination? We hypothesized that (H1) *E. gmelinii* shows plasticity in the life history traits in response to the different environment resulting from rainfall treatments; (H2) the effect of maternal environment on offspring germination; (H3) likely future scenarios of increasing cool-season precipitation and large rainfall events will enhance the growth of winter annuals in temperate desert.

## Materials & Methods

### Study site

**Figure 1:**
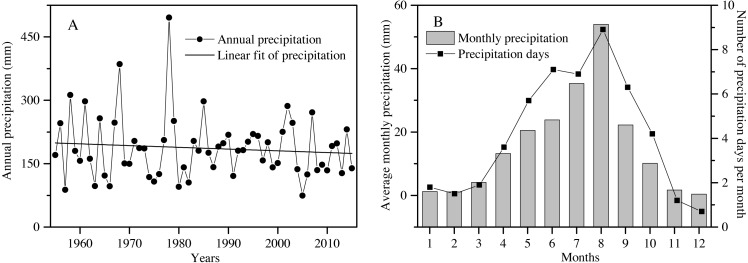
The characteristics of precipitation variation during the past 60 years (1955 to 2015) in the Shapotou region (from Shapotou Station meteorological data). Annual precipitation amount (A), average monthly precipitation and number of precipitation days per month (B).

The study area is located at the Shapotou Desert Research and Experimental Station (Shapotou Station) at the southeastern edge of the Tengger Desert (37°32′N, 105°02′E). The annual mean temperature is 9.6 °C, the minimum temperature is −25.1 °C, and the maximum temperature is 38.1 °C. Over a 60-year period, the rainfall amount slightly decreased, with great interannual fluctuation (Fig. 1A). The mean annual precipitation is 186.2 mm, of which nearly 90% falls between April and September. The mean number of precipitation days (days of precipitation ≥0.1 mm ) was 50 (Fig. 1B), and approximately 80% of the rain days were less than 5 mm of rainfall accumulation throughout the year (Zhang et al., 2016).

To protect the Baotou-Lanzhou railway line from sand burial, a nonirrigated vegetation protection system was established in 1965 by Shapotou Station. To extend the research to vegetation successional processes and water cycles in the restored vegetation area, the Water Balance Experimental Field (WBEF) was established by the Shapotou Station in April 1989. The WBEF was constructed by first levelling sand dunes, then erecting sand barriers using 1 × 1 m wheat-straw checkerboards, and finally planting xerophytic shrubs *Artemisia ordosica* Krasch. and *Caragana korshinskii* Kom. in different years (Zhang et al., 2016). In the artificial vegetation sand-fixing area of the Shapotou region, winter annual *E. gmelinii* is a pioneer and dominant herbaceous species at the early stage of dune fixation, which plays a vital role in preventing wind erosion and maintaining sand fixation during the spring and early summer.

### Experimental design

In our study area, except for two extremely high-precipitation years (1968 and 1978) and two extremely low-precipitation years (1957 and 2005), the interannual variation in the amount of precipitation was from 51 to 159% of the average annual precipitation (186.6 mm) over the past 60 years (Fig. 1A). To understand the effects of rainfall amount and frequency on plant survival, growth and reproduction of overwintered seedlings from April to July, an array of 25 rainfall treatments (5 total amounts × 5 frequencies) was established in our experiment. Each treatment was replicated 12 times (300 total pots). Accordingly, a gradient of different rainfall amounts was established to approximate the observed variation in rainfall in the total amounts of 150, 125, 100, 75 or 50% quantity of the mean total rainfall from April to July (92 mm) corresponding to 138, 115, 92, 69 and 46 mm, respectively (Fig. 1B; Table 1). In addition, the mean frequency of rainfall was one rainfall event every 5.2 days in our study area from April to July (Fig. 1B). We assumed that the frequency will continue to change in the future. Thus, a gradient of rainfall frequencies was established within each rainfall amount treatment, in which the plants were watered every 3 days, 5 days, 10 days, 15 days or 30 days (Table 1).

**Table 1:** Total amounts and frequencies of rainfall events for treatments in the experiment.

Quantity change (%)	Rainfall event size for each interval (mm)					Total amount (mm)
	3-d	5-d	10-d	15-d	30-d	
150	3.45	5.75	11.50	17.25	34.50	138
125	2.88	4.79	9.58	14.38	28.75	115
100	2.30	3.83	7.67	11.50	23.00	92
75	1.73	2.88	5.75	8.63	17.25	69
50	1.15	1.92	3.83	5.75	11.50	46

The experiment was conducted in a complete rainout shelter (12 × 6 m) consisting of a steel frame, which was constructed on level sandy soil in the WBEF of Shapotou Station. The rainout shelter was assembled to obtain a maximum shelter height of 2.1 m angled to a minimum height of 1.8 m. Roofing consisted of clear polycarbonate panels that eliminated ultraviolet radiation but transmitted 90% of visible light. The shelter sides remained open to maximize air movement and minimize temperature and relative humidity differences from those in the ambient environment. All pots were buried about 18-cm-deep into the sandy soil beneath the rainout shelter to avoid damage to plants that may occur at low temperatures in winter. The simulated rainfall treatments lasted for 110 days between 3 April and 20 July 2018, since this is the time to complete the vegetative and reproductive growth for *E. gmelinii* in the natural habitat. Water was added to pots by measuring the given amount in a beaker and then gently and uniformly pouring it over the sand. For the 3–15-day interval treatments, all water for a given event was applied on 1 day. For the 30-day interval treatment watering was successively distributed over 1–3 days according to the appropriate water amounts to reduce water leakage and runoff.

In our study area, *E. gmelinii* seedlings emerge in summer and autumn, and overwinter as rosettes. The overwintered seedlings vegetative and reproductive growth mainly utilize spring and early summer (from April to July) rainfall of the following year. To insure uniformity in overwintered seedlings for the controlled rainfall experiment, *E. gmelinii* seeds were collected in late June 2017 from a natural population in the vegetation sand fixation area in the WBEF and were stored in paper bags under ambient room conditions until use in the laboratory. In late August 2017, seeds were sown in the pots (height × top diameter × bottom diameter = 20 × 26 × 18 cm; with drainage holes at the bottom) filled with approximately 10 kg of sand. Ten seeds were sown into each pot. The pot size used in the experiment was chosen on the basis of the plant size and root length in our study system. The sandy soil was taken from 50-cm-deep underground in the natural habitat of *E. gmelinii*. In addition, the nonwoven cloth was placed on the bottom of the pots to allow water to permeate but prevent plant roots from stretching out of the pot. During the seed germination period, the soil was watered daily to field capacity to ensure the successful establishment of seedlings. To prevent variation in initial seedling size, seedling emergence was checked daily. Most of seedlings emerged on the fifth day; a few seeds germinated on the third and fourth days, and these seedlings were removed and discarded. In addition, at the two-leaf stage (i.e., 2 weeks after emergence), five seedlings of the same size in each pot were kept for overwintering. Furthermore, to ensure that the seedlings could survive to overwinter successfully, all pots were watered to field capacity every 5–7 days between September and October. Additionally, based on the mean monthly precipitation over 60 years (Fig. 1B), the amount of water applied to simulate rainfall was 2 mm, 2 mm and 5 mm on November 10, January 5 and March 15 in the following year, respectively. In late March 2018, new leaves appeared on the overwintered seedlings, and to prevent variation in initial seedling size, seedlings of the same size in each pot were kept (one plant per pot), and the others were removed from the pots.

To determine the soil water content of the 0–20 cm soil depth in the pots, we prepared 3 pots per treatment with sand but no plants and employed identical rainfall treatments as those applied to the pots with plants. We collected three soil samples (soil cores with diameter of 3 cm) of 0–20 cm from the pots without plants per treatment daily for up to 14 days between 3 and 16 May. These samples were then immediately placed in soil sample cans, and the moisture content was measured by the oven-drying method.

### Measurements

The plant mortality was recorded every three days in all treatments from April to July. Our field investigation of *E. gmelinii* population for three years (2016 to 2018) showed that less than 30% of seedlings could successfully overwinter in our study area (Wang et al., 2019b). Thus, survival of overwintered seedlings is a key factor for affecting population dynamics of *E. gmelinii*. We measured the height of each plant (height_1_) in early April 2018 following the first time the water treatments were applied and measured the height (height_2_) again in late April. The same measurements were conducted in May. These dates correspond to rapid periods of vegetative growth among these plants. For each surviving plant, we calculated the relative height increase (Δ_height_) over a 1-month period as ln (height_2_) − ln (height_1_).

On July 20 2018, all seeds were mature and the plants were completely harvested from the pots. Individual plant height was measured and the number of capitula per plant was recorded. Moreover, since the seed maturity dates were different under different rainfall treatments, we covered the capitula using poly-organza bags (40 fine-mesh) before the seeds detached to prevent seed loss. All seeds were collected from the plants and counted. The infructescences (without seeds), leaves, stems and roots (washed free of sand) of each survival plant were detached and weighed separately after drying at 75 °C for 48 h. Seeds were placed for one month in the laboratory to dry naturally and then germination tests were performed in a separate experiment (see below). Once dry, all parts were weighed using an electronic-balance (0.001 g). All seeds per plant were weighed and the mass of 100 seeds was determined to analyze the difference in seed mass among different treatments. Total biomass was calculated as the sum of dry mass of seeds and infructescences, leaves, stems and roots of each plant. The root/shoot ratio was computed as the root dry mass to shoot dry mass. Reproductive effort was calculated as the ratio of total reproductive (seed + infructescences) mass to total biomass per plant (Harper & Ogden, 1970).

### Seed germination of offspring

Mature seeds were stored in paper bags under ambient room conditions until use. Germination experiments started on 20 August 2018. Germination tests were conducted in incubators under optimum conditions (12 h light/ 12 h dark, 30/20 °C) for *E. gmelinii* seed germination (Wang et al., 2019a). Three replicates of 20 seeds each were sown on two layers of Whatman No. 1 filter paper in 9 cm-diameter glass Petri dishes and moistened with distilled water. Seeds were monitored and watered (if necessary) every day. Germination was considered as protrusion of the radicle (∼2 mm long), and these seeds were discarded. The viability of ungerminated seeds was determined as follows: seeds were cut in half and soaked in 0.5% 2,3,5-triphenyl tetrazolium chloride (TTC) at a constant 25 °C for 3 h; cotyledons that were stained red were considered viable. Final germination percentages were based on the number of viable seeds.

### Data analysis

All statistical analyses were performed with SPSS version 16.0 (SPSS Inc., Chicago, IL, USA). Effects of rainfall amount on seedling survival were analyzed with a chi-square test of independence. The chi-square analysis was also used to test whether survival varied with rainfall frequency across amount treatments. Two-way analysis of variance (ANOVA) was carried out to compare the effects of the total amount and frequency of rainfall and their interaction on components of growth (height, biomass and root/shoot ratio), reproductive traits (capitula number, seeds number, seed mass and reproductive effort) and offspring seed germination. Significant interactions would indicate that the response of a trait to the total amount of rainfall was highly dependent on the frequency of rainfall. The relative magnitudes of the effects (the effect size) were estimated according to the partial eta squared [*η*${}_{\mathrm{p}}^{2}$ = SS effect/(SS effect + SS error), SS = sum of squares], which measures the relative explanatory power of the effect of the independent variable on the dependent variable (Burns et al., 2008). Data were log- (i.e., seeds and capitula number) or arcsin-transformed (i.e., seed germination) before analysis when required to satisfy assumptions of ANOVA. Non-transformed data appear in all figures. The averages were compared by protected least significant difference tests (LSD) at the 5% level of significance. Five treatments in which all plants died were discarded from the analysis.

## Results

**Figure 2:**
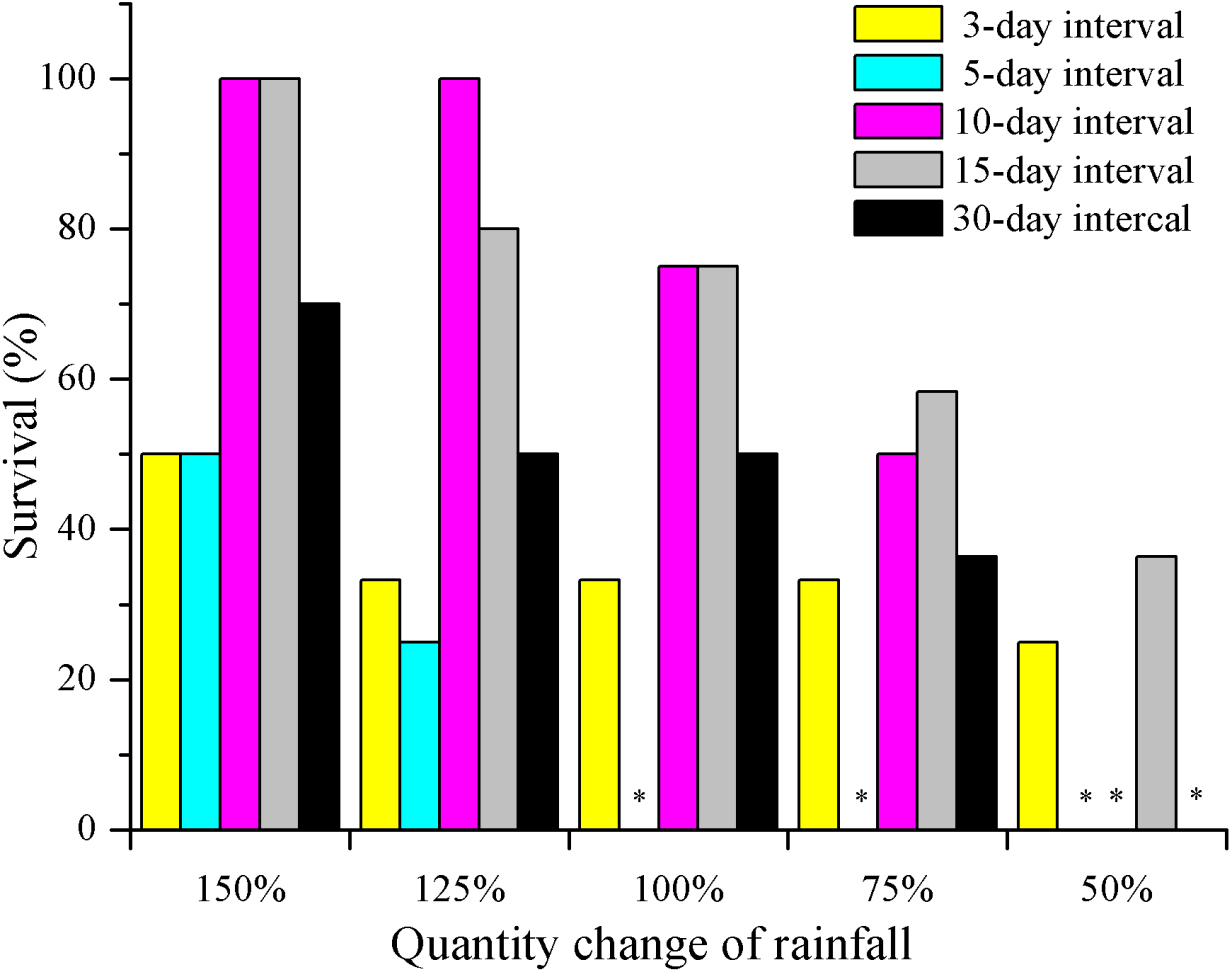
The effects of rainfall variation (amounts and frequencies) on *E. gmelinii* survival. The significant differences were tested using the chi-square test of independence. Overall, there were significant differences among frequencies within each amount (Chi-square for 150% = 15.6, 125% = 20.4, 100% = 19.2, 75% = 11.6 and 50% = 10.3, both have a *P* < 0.01). Except for 3-day interval treatment (Chi-square for 3-d = 1.8, *P* = 0.843 > 0.05), the differences among rainfall amounts within each frequency were significant (Chi-square for 5-d = 14.9, 10-d = 39.0, 15-d = 11.9 and 30-d = 14.2, both have a *P* < 0.01). Asterisks indicate that no data are available due to the death of all plants.

Seedling survival of *E. gmelinii* decreased with decreasing amounts of rainfall regardless of the frequency (Fig. 2). Moreover, except for 3-day interval treatment, the differences among rainfall amounts within each frequency were significant (*P* < 0.01, Chi-square test). Survival in the high-frequency treatment (3- and 5-day intervals) was low and less than 50% across all rainfall amount treatments. Especially under the 5-day interval treatment, all plants died (*n* = 12) before seed production under 50–100% rainfall treatments. In the 10- and 15-day interval treatments, high survival (80–100%) occurred under 150% and 125% rainfall. Moreover, 36.4% (*n* = 11) of seedlings could survive to produce seeds in the 15-day interval treatment under extremely low water availability (50%). In the low-frequency treatment (30-day interval), the survival rate was 50–75% under 100–150% of rainfall amount. Overall, there were significant differences among frequencies within each amount (Chi-square for 150% = 15.6, 125% = 20.4, 100% = 19.2, 75% = 11.6 and 50% = 10.3, both have a *P* < 0.01).

**Figure 3:**
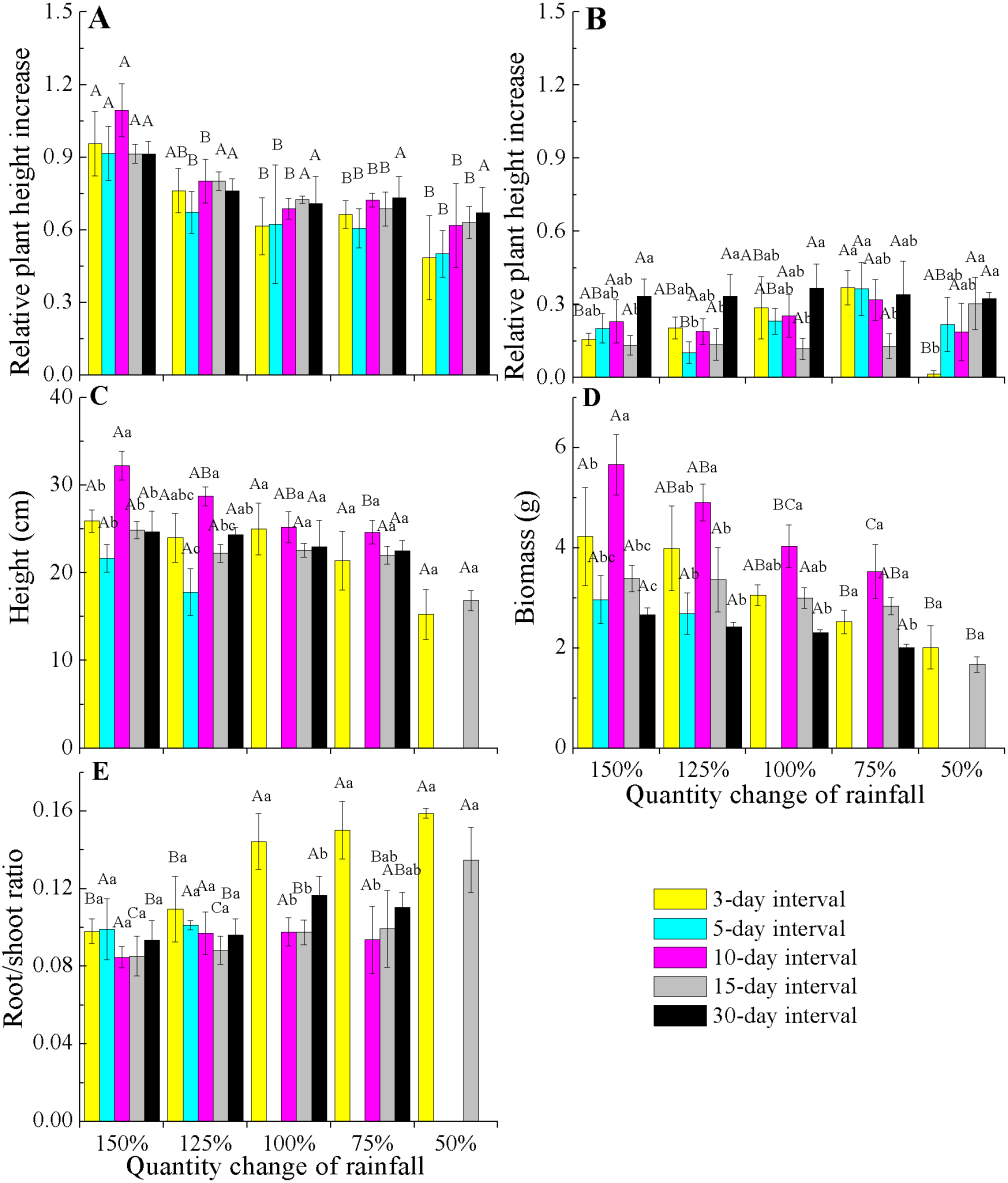
Effects of rainfall variation (amounts and frequencies) on plant growth traits (mean ± SE) in *E. gmelinii*. Relative plant height increase (Δ height) in April (A) and May (B), height (C), total biomass (D)and root/shoot ratio (E). Bars with the same upper-case letters indicate nonsignificant differences among rainfall amounts within each frequency and those with the same lowercase letters show nonsignificant differences among frequencies within each amount.

When comparing the rates of plant height increase (Δ height) in *E. gmelinii* in April and May, the results showed that rapid growth in plant height occurred in April (Figs. 3A and 3B). Also in April, the Δ height under the 150% rainfall was significantly (*P* < 0.05) higher than that in other rainfall amounts under all rainfall frequency treatments. However, there were no significant (*P* > 0.05) differences in Δ height under 50–125% rainfall. Similarly, no differences were detected due to changes in rainfall frequency (Fig. 3A). The effects of the different amounts and frequencies of rainfall were significant for plant growth traits (Figs. 3C–3E). With decreasing amounts of rainfall, plant height and biomass significantly decreased, but the root/shoot ratio increased. Across frequencies within each rainfall amount, plant height and biomass were largest at the 10-day interval. Under the high rainfall treatments (150% and 125%), the effects of the different rainfall frequencies on height were significant, while under the low rainfall treatments (50–100%), the effects were not evident. The root/shoot ratio at 3-day interval was significantly higher than that at other rainfall frequencies under 75–100% rainfall, while the effects of the different frequencies on the root/shoot ratio was not significant under 150% and 125% rainfall. Moreover, the effect of rainfall frequency on the root/shoot ratio depended on the rainfall amount to some degree (interactions, *F* = 2.554, *P* < 0.05, *df* = 111; Table 2).

**Table 2:** Results of two-way ANOVAs for the effects of rainfall amount and frequency on *E. gmelinii*. Explained variation (effect size) is given by partial eta squared values (*η*2). The *P*-values in bold indicate that the differences were significantly (*P* < 0.05).

	Amount				Frequency				Amount × frequency				Error
	df	F	P	*η*${}_{\mathrm{p}}^{2}$	df	F	P	*η*${}_{\mathrm{p}}^{2}$	df	F	P	*η*${}_{\mathrm{p}}^{2}$	df
Height	4	12.479	**0.000**	0.310	4	14.735	**0.000**	0.347	11	1.082	0.383	0.097	111
Biomass	4	5.098	**0.001**	0.155	4	10.091	**0.000**	0.267	11	1.026	0.428	0.092	111
Root/shoot ratio	4	6.637	**0.000**	0.193	4	3.697	**0.007**	0.118	11	2.554	**0.006**	0.202	111
Number of capitula per plant	4	12.362	**0.000**	0.308	4	14.070	**0.000**	0.336	11	2.709	**0.004**	0.212	111
Number of seeds per plant	4	4.604	0.221	0.050	4	6.939	**0.000**	0.226	11	0.919	0.167	0.124	111
Seeds mass	4	11.830	**0.000**	0.299	4	5.528	**0.000**	0.166	11	2.306	**0.014**	0.186	111
Reproductive effort	4	50.602	**0.000**	0.646	4	34.594	**0.000**	0.555	11	1.228	0.277	0.109	111
Offspring seed germination	4	8.919	**0.000**	0.471	4	13.734	**0.000**	0.579	11	4.376	**0.000**	0.546	40

**Figure 4:**
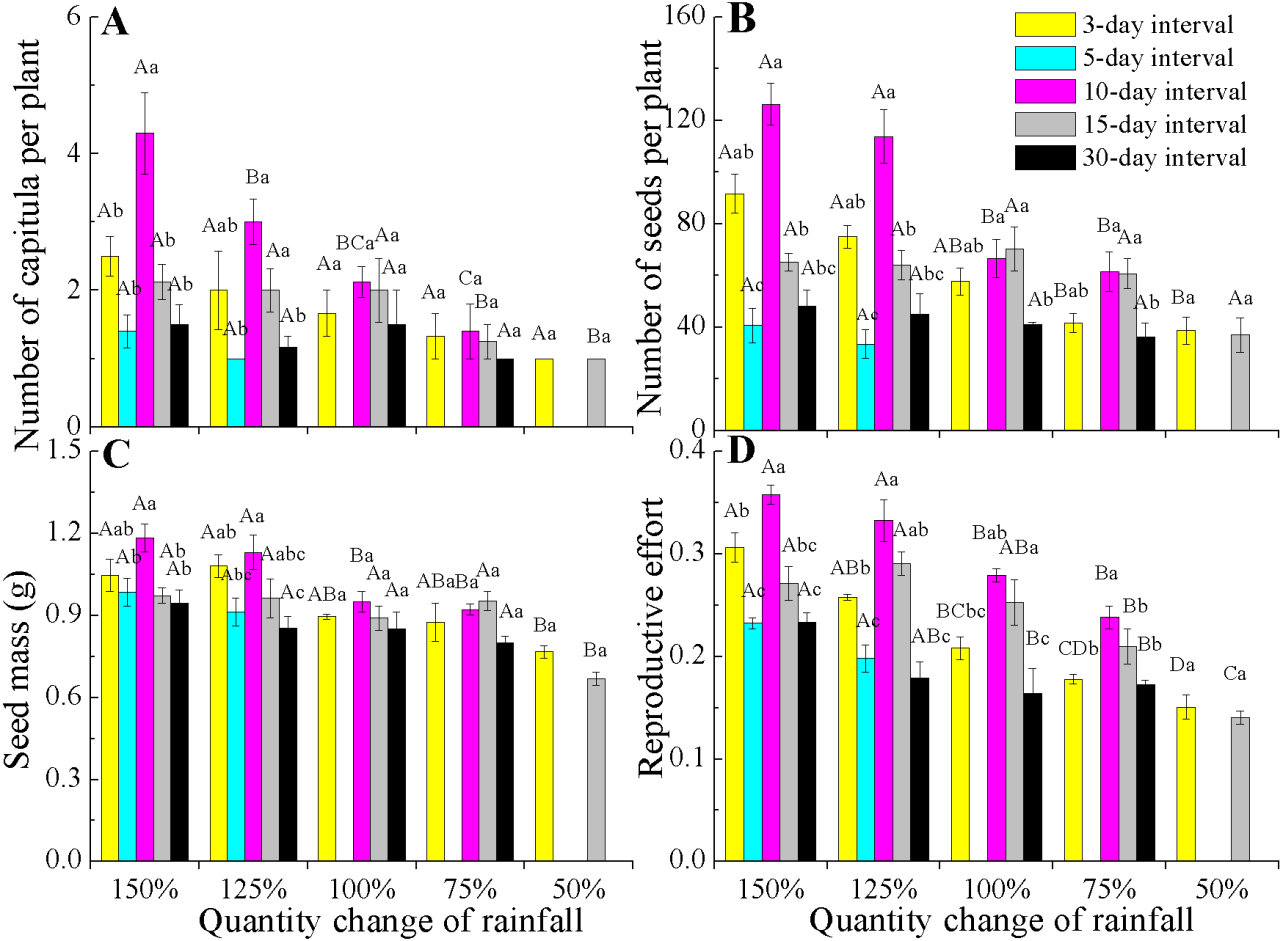
Effects of rainfall treatments on reproductive traits (mean ± SE) in *E. gmelinii*. Number of capitula per plant (A), number of seeds per plant (B), seed mass (C) and reproductive effort (D). The traits of seed mass and reproductive effort were calculated on a dry mass basis. Bars with the same upper-case letters indicate nonsignificant differences among rainfall amounts within each frequency and those with the same lower-case letters show nonsignificant differences among frequencies within each amount.

All reproductive traits also significantly differed among the various rainfall amount and frequency treatments (*P* < 0.05, Table 2). The capitula number and seed mass associated with the different rainfall amounts were highly dependent on rainfall frequencies (interactions, *P* < 0.05). Under 150% and 125% rainfall, all reproductive traits were the largest at the 10-day interval, but they decreased at 50–100% of rainfall. Notably, under 50–100% rainfall, seed mass and the number of capitula and seeds were stable across the different amounts and frequencies (Figs. 4A–4C). However, the reproductive effort significantly decreased with decreasing rainfall amount regardless of frequency (Fig. 4D).

**Figure 5:**
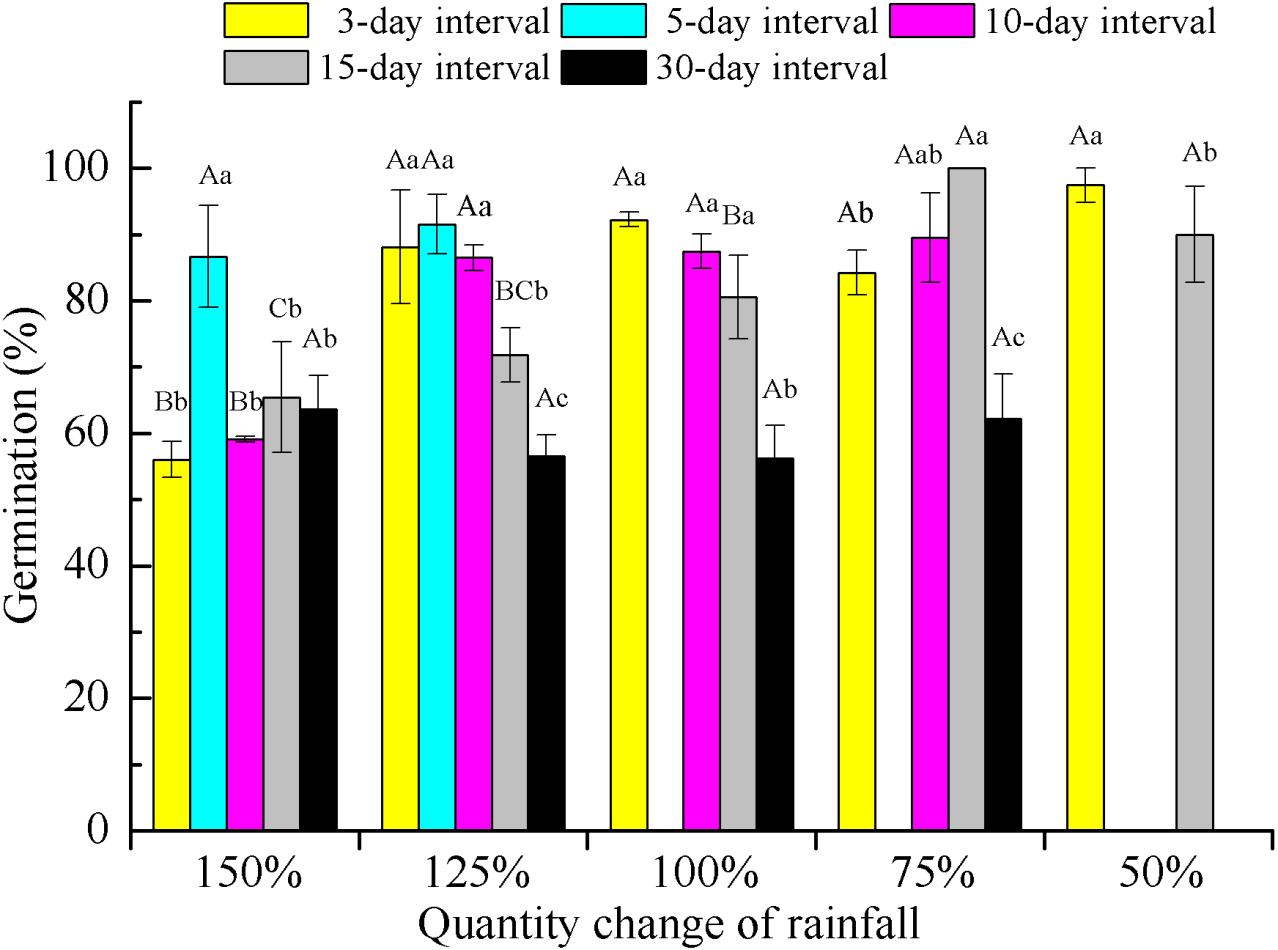
Germination percentage (mean ± SE) of *E. gmelinii* seeds from plants grown under different amounts and frequencies of rainfall. Seeds were incubated for 2 weeks in light/dark at an alternating temperature (30/20 °C, 12/12 h). Bars with the same upper-case letters indicate nonsignificant differences among rainfall amounts within each frequency and those with the same lower-case letters show nonsignificant differences among frequencies within each amount.

Two-way ANOVA of the effects of rainfall pattern on offspring germination demonstrated that rainfall amount, frequency and their interaction had significant (*P* < 0.001) effects on the final germination percentage (Table 2). Rainfall frequency explained more variation than amount for germination (57.9 vs 47.1%). With the exception of the extreme low-frequency treatment (30-day interval), offspring germination showed an increasing trend with a decrease in rainfall amount within each rainfall frequency. At a rainfall frequency of 30-day intervals, the difference in rainfall amount on germination was not significant (*F* = 0.558, *P* > 0.05, *df* = 3) and germination was significantly (*P* < 0.05) lower than that found for the other frequencies under 75–125% rainfall (Fig. 5).

**Figure 6:**
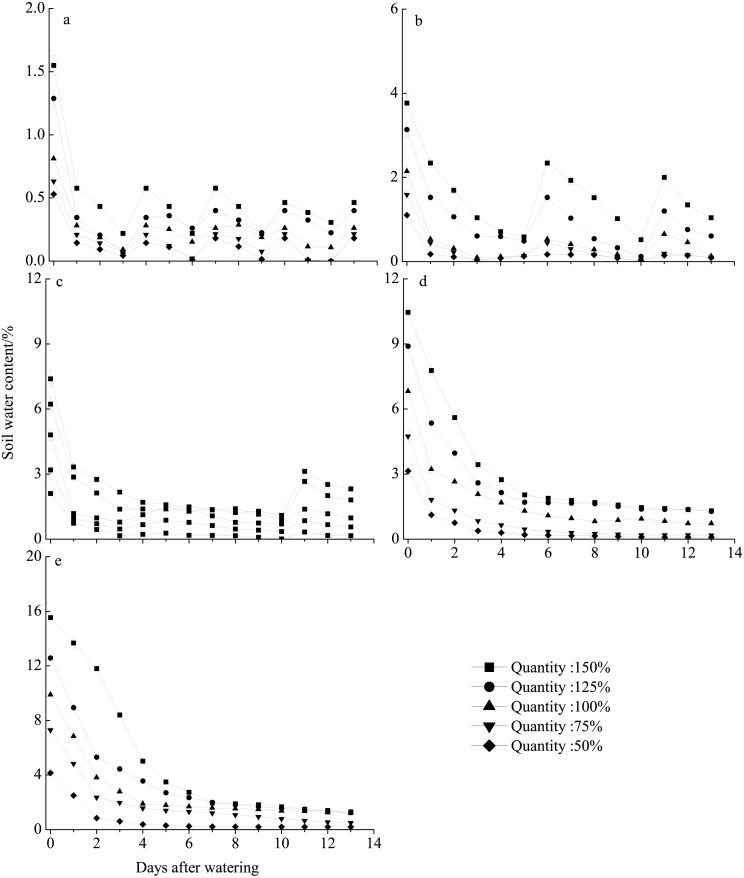
Soil water content dynamics (mean ± SE) in the different rainfall treatments. The varied in terms of total rainfall amount (50–150%) and frequency, with watering occurring every 3 (A), 5 (B), 10 (C), 15 (D), or 30 (E) days.

The different rainfall amount and frequency treatments directly led to differences in soil water content (Fig. 6). Pots receiving the high rainfall treatment (100–150%) maintained greater soil moisture than those receiving the low rainfall amount (50–75%) for approximately 2 weeks. The sand water content in all treatments was highest immediately following watering and then gradually decreased. In all frequency treatments, the sand water content decreased to nearly 0% before the next watering at 75% and 50% of rainfall amount. For the 10–30 day intervals, the soil water content rapidly decreased in the first 5 days after watering and then remained relatively stable with the sand water content being greater than 1% during our observational period under 100–150% rainfall.

## Discussion

Understanding the mechanisms of plant survival, growth and reproduction in response to rainfall pattern changes is critical to predicting plant population persistence under altered climate regimes. In semiarid and arid regions, ecosystems on sandy soils can be particularly sensitive to rainfall changes, partly due to the low water-holding capacity of the soil (Huang et al., 2017). Desert annual herbs respond very rapidly to rainfall changes throughout the whole growth period, and species show differential responses to unpredictable precipitation (Chen et al., 2019b). In our study, *E. gmelinii* seedling survival, plant growth and reproduction showed mostly consistent responses along the gradient of rainfall amounts and along the frequency gradient. Under each rainfall amount, the optimum rainfall frequency for plant growth and reproduction was a 10-day interval. Furthermore, *E. gmelinii* showed strong plasticity in measured traits in response to rainfall variations. Additionally, seed germination of offspring tended to increase with increasing aridity, suggesting that a maternal effect may have occurred. Therefore, variations of rainfall amount and frequency will affect plant population regeneration of the winter annual on sand dunes.

In this study, seedling survival rate was highest (100%) at frequencies of 10- and 15-day intervals under 150% and 125% rainfall (Fig. 2). These rainfall patterns were associated with greater water infiltration of the soil water, and high soil moisture persisted longer than that under the other treatment, especially at deeper soil depths, where evaporation was low (Fig. 6). Nevertheless, under the 150% rainfall, the survival rate was less than 50% at high frequencies (3- and 5-day intervals), as these treatments entailed consecutive sequences of small precipitation pulses that caused the soil to be relatively dry because the individual watering amounts were very low. Under the extreme low frequency (30-day interval) and amount (50%) of rainfall, a small number of *E. gmelinii* seedlings were able to survive to produce seeds, most likely because the plants are adapted to the characteristic inter- and intra-annual climatic variability that occurs at arid sites (Jump & Penuelas, 2005). Another reason may be that *E. gmelinii* germinates in summer and autumn, and the overwintering seedlings had reached a certain size with well-developed roots (length of 10 to 20 cm), which could survive under a low amount and frequency of rainfall in spring. The results could explain why some desert plants, many of which have deep root systems, show the greatest response to low-frequency and large rainfall events (Shan et al., 2018). Thus, winter annuals would be expected to increase and improve reproductive success with variable precipitation under the future climate.

Most species tolerate short-term climate variability through phenotypic plasticity (Jump & Penuelas, 2005), especially in short-lived organisms living in harsh ecosystems. Our study show that rainfall changes had marked impacts on *E. gmelinii* growth and reproduction. The growth and reproductive traits remarkably decreased with a declines in rainfall amount under each rainfall frequency, but the opposite trend was observed for the root/shoot ratio (Figs. 3 and 4). Similarly, some water manipulation experiments in arid and semiarid ecosystems showed that reductions in the amount of rainfall usually limit plant growth and/or seed production, whereas an increased water supply has the opposite effect (Breen & Richards, 2008; Volis, Ormanbekova & Yermekbayev, 2015; Mojzes et al., 2018). The response of *E. gmelinii* root/shoot ratio to rainfall amount was consistent with that of plants growing in arid regions, in which increased allocation to roots may be advantageous for capturing limiting soil resources (Padilla et al., 2013; Gao et al., 2015; Shan et al., 2018; Chen et al., 2019a). Moreover, under high amounts of rainfall (150% and 125%), plant growth and reproductive traits under the rainfall frequency of 10-day interval were significantly higher than those observed in association with other frequencies (Figs. 3 and 4). These results indicate that *E. gmelinii* shows strong plasticity in growth and reproduction in response to rainfall variation, which enhances its ability to survive and reproduce in the unpredictable environments of arid regions. However, under low rainfall (50–100%), plant height, biomass, capitula number and seed mass were stable across all frequencies, suggesting that plant exhibit low plasticity in response to rainfall frequencies when a low amount of rainfall occurs. Thus, the effects of rainfall frequency on plant growth and reproduction depended on the amount of rainfall to some degree.

Reproductive effort of *E. gmelinii* significantly decreasing with a decline in rainfall amount across all rainfall frequencies (Fig. 4D). Moreover, rainfall amount and frequency explained more variation for reproductive effort than other measured traits (Table 2), indicating that the plasticity of reproductive effort is more sensitive to the rainfall variations. Similarly, the reproductive effort of the winter annual *Brachypodium distachyon* was also found to decrease with increasing aridity (Aronson, Kigel & Shmida, 1993). However, some studies have frequently claimed that reproductive effort is an invariant characteristic of a species or population that remains constant even when plants are exposed to various stresses (Harper & Ogden, 1970; Hickman, 1977; Abrahamson, 1979; Schlichting & Levin, 1984; Angert et al., 2010). Indeed, other published works (Monson & Szarek, 1981; Marshall, Levin & Fowler, 1986; Aronson, Kigel & Shmida, 1990; Aronson, Kigel & Shmida, 1993) and our results clearly indicate that reproductive effort is a dynamic component of annual species’ adaptive life history strategies in the desert environment and is readily affected by changes in rainfall. A previous study showed that seed size is often the least plastic component of reproductive yield within a species (Harper & Ogden, 1970). The effects of rainfall variations on the reproductive components were mainly due to changes in seed number and much less to variation in seed size. Furthermore, our results showed that rainfall frequency explained more variation than rainfall amount for capitula and seeds number (Table 2), suggesting that the plasticity of reproduction traits to the change of rainfall amount is lower than to frequency. Thus, it can be inferred that the development of plant population is highly susceptible to future rainfall amount changes.

In our study, we also found that seed germination of *E. gmelinii* offspring increased with decreasing rainfall amount at a given frequency, with the exception of the 30-day rainfall interval (Fig. 5), indicating that maternal effects may have occurred. Similar results have been reported in rainfall manipulation experiments with annual species (Karimmojeni et al., 2014; Gao et al., 2015). Seed dormancy imposed by drought during seed development usually decreases dormancy and increases germinability (Fenner, 1991). In contrast, other studies have reported similar or higher seed germination of offspring in response to better water conditions in the maternal environment (Breen & Richards, 2008; Pias et al., 2010; Yang et al., 2011). However, Mojzes et al. (2018) showed that offspring seed germination in the winter annual grass *Secale sylvestre* was not influenced by the environmental conditions associated with their mother plants. The offspring seed germination of *E. gmelinii* was negatively correlated with high reproduction effort among their mother plants, which could prevent most of the seed germination occurring with a single year and contribute to the formation of a persistent soil seed bank. Moreover, low seed germination could prevent high levels of competition among siblings when a large number of seeds are produced in wet environments (Chen et al., 2019b). Higher seed germination might allow some plants survival when only a few seeds are produced in the dryer environments (Wagmann et al., 2012). The seed germination of *E. gmelinii* offspring under 30-day rainfall intervals was significantly lower than that under other frequencies at 75–125% rainfall, which may be due to the low seedling survival under these conditions. These results also demonstrate the maternal effects associated with rainfall frequency on offspring germination.

## Conclusions

In temperate zones, patterns of rainfall are currently changing, with the occurrence of extreme rainfall events, increasing in rainfall intervals and changes in seasonality (less summer and more cool-season precipitation), and these patterns are expected to change further under global warming (IPCC, 2013). Climate change is known to influence seedling survival and fecundity in annual plants, which can strongly affect population persistence and community dynamics in arid systems (Levine, Mceachern & Cowan, 2008). The growth and reproduction stages of *E. gmelinii* mainly occur in the cool and dry seasons, and less than 40% of the precipitation distributes in the spring, autumn and winter in the Shapotou region (Fig. 1B). Our results show that increased rainfall in spring and early summer significantly improved seedling survival, growth and reproduction, which all exhibited a greater response to low frequency (10-day interval) and large rainfall events. In addition, the variability of reproductive effort in response to rainfall variation is a critical component of life history strategies in *E. gmelinii* in unpredictable desert environments. Further, we found that variations of rainfall amount and frequency in maternal environment could influence the germination behaviors of offspring, which can reduce the risk of germination failure and maintain the population. By and large, these results indicate that the plastic response of the growth and reproduction of *E. gmelinii* to rainfall fluctuations shows strong adaptation to the currently unpredictable environment as well as the increased unpredictability under climate change. Therefore, this species has multiple life history strategies for dealing with unpredictable environmental, which should be very adaptive under the expected future scenarios of increasing cool-season precipitation and large rainfall events. Simultaneously, our findings highlight the inherent complexity in predicting desert ecosystem responses to fluctuations in precipitation, and provide a mechanistic understanding of projecting plant population dynamics under global change.

##  Supplemental Information

10.7717/peerj.8159/supp-1 File S1 Raw data

## Additional Information and Declarations

### Data Availability

The following information was supplied regarding data availability: The raw measurements are available as a Supplemental File.
